# Cellular Internalization and Exiting Behavior of Zwitterionic 4-Armed Star-Shaped Polymers

**DOI:** 10.3390/molecules28114479

**Published:** 2023-06-01

**Authors:** Yuta Yoshizaki, Tomohiro Konno

**Affiliations:** Graduate School of Pharmaceutical Sciences, Tohoku University, Sendai 980-8578, Japan; yuta.yoshizaki.c5@tohoku.ac.jp

**Keywords:** phospholipid polymer, amphiphilic polymer, star polymer, atom transfer radical polymerization, cellular internalization, cell shuttle

## Abstract

The zwitterionic phospholipid polymer poly(2-methacryloyloxyethyl phosphorylcholine-*co*-*n*-butyl methacrylate) (PMB) is amphiphilic copolymer, and it has been reported to directly penetrate cell membranes and have good cytocompatibility. Conventional PMBs are linear-type random copolymers that are polymerized by a free radical polymerization technique. In contrast, star-shaped polymers, or simple branched-type polymers, have unique properties compared to the linear types, for example, a viscosity based on the effect of the excluded volume. In this study, a branched architecture was introduced into a PMB molecular structure, and a 4-armed star-shaped PMB (4armPMB) was synthesized by an atom transfer radical polymerization (ATRP) technique known as living radical polymerization. Linear-type PMB was also synthesized using ATRP. The effects of the polymer architecture on cytotoxicity and cellular uptake were investigated. Both 4armPMB and LinearPMB were successfully synthesized, and these polymers were verified to be water soluble. Pyrene fluorescence in the polymer solution indicated that the architecture had no effect on the behavior of the polymer aggregates. In addition, these polymers caused no cytotoxicity or cell membrane damage. The 4armPMB and LinearPMB penetrated into the cells after a short incubation period, with similar rates. In contrast, the 4armPMB showed a quicker back-diffusion from the cells than that of LinearPMB. The 4armPMB showed fast cellular internalization and exiting behaviors.

## 1. Introduction

Recently, nucleic acid-based and protein-based drugs have been launched for intractable and emerging infectious diseases [1,2]. CRISPR-Cas9 is a strong biomedical tool for genome editing that requires the introduction of nucleic acids and nuclease proteins into cells [3,4]. A high molecular weight compound, such as a nucleic acid or protein, is taken up by cells via endocytosis. The majority of the molecules incorporated into the cells are completely degraded in the endosome/lysosome [5]. Accordingly, the development of a drug carrier for cytosolic delivery [6] is important to achieve the desired effect of the nucleic acid or protein.

Numerous drug carriers have been studied for many years, including viral vectors [7], lipid nanoparticles [8], polymer micelles [9], and inorganic nanoparticles [10]. Viral vectors can cause unexpected immune responses [11], although their immunogenicity has been reduced through the endeavors of researchers. Non-viral vectors implement endosomal escape using membrane fusion, which is known as the proton sponge effect [12,13,14]. Many mechanisms of endosomal escape utilize cationic lipids, polymers, and peptides, which can cause inflammatory reactions [15,16,17]. This inflammatory effect functions as an immunological potentiator and adjuvant in vaccines, although the development of carriers that are unaffected by the immune system is also important in biomedical applications.

Both 2-methacryloyloxyethyl phosphorylcholine (MPC) and *n*-butyl methacrylate (BMA) can be copolymerized via conventional radical polymerization to give poly(MPC-*co*-BMA) (PMB), which exhibits prominent cytocompatibility. PMB is a zwitterionic polymer derived from MPC units bearing a phosphorylcholine group in the side chain, and a water-soluble or insoluble polymer depending on its molecular weight and copolymer composition [18,19]. The water-soluble PMB can solubilize hydrophobic drugs [20,21] and directly penetrate cell membranes [22]. PMB with an active ester unit successfully delivered oligonucleotides, called molecular beacons [23]. These polymers are linear-type random polymers, and adjustment of the copolymer composition is effective for the development of useful polymer materials. The polymer molecular architecture is an important factor in determining the performance of polymers. A branched polymer with a single branched point is called a star polymer. Star polymers and linear polymers exhibit different properties, such as viscosity, hydrodynamic diameter, and glass transition temperature, even if the two polymers have the same monomer composition [24,25,26].

In the development of drug carriers, many studies focused on how to deliver and release the cargo inside the cells. In contrast, many studies took little notice of how carriers themselves followed the fate in the cell after releasing the cargo. PMB was internalized to cells, and then PMB could exit from cells because PMB directly penetrated the plasma membrane. This property to exit from cells is regarded as back-diffusion from cells. The back-diffusion may help to enhance the performance of drug carriers. For instance, the back-diffusion may enhance nucleic acid delivery carriers. These carriers require electrostatic interaction between nucleic acid and carriers, and nucleic acid is condensed by molecules composing carriers, such as polycation [27]. This condensed state of nucleic acid impedes the transcription and/or translation process, and then it impedes gene expression [28]. In this case, carriers exhibiting the back-diffusion may not impede the process of gene expression. Investigation of the back-diffusion could be helpful in the field of drug delivery.

**Figure 1:**
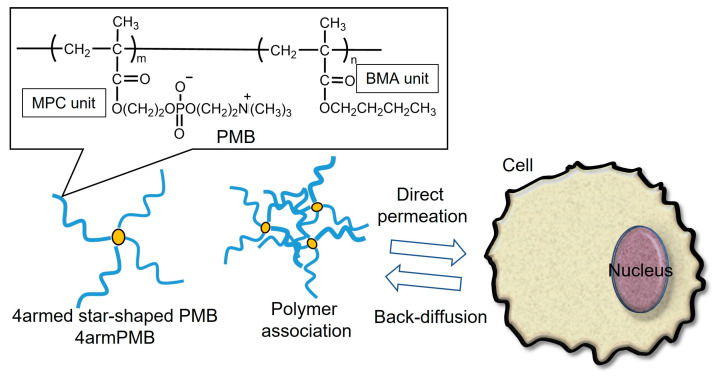
Schematic illustration of 4-armed star-shaped poly(MPC-*co*-BMA) (4armPMB) permeate plasma membrane bidirectionally.

In this study, we synthesized star-shaped zwitterionic polymers with precisely defined structures and investigated their cellular internalization and exiting behavior (Figure 1). The 4-armed-star polymers were synthesized via activator regenerated by electron transfer (ARGET) atom transfer radical polymerization (ATRP). ARGET-ATRP is a living radical polymerization method that has the advantage of reducing the amount of copper catalyst required [29]. Linear-type and 4-armed star-type PMB were synthesized by changing the initiator, and the cell membrane damage and cytotoxicity of these polymers were investigated using cultured cells. We then investigated the cellular internalization of the polymers and the back-diffusion of polymers from the cells.

## 2. Results and Discussion

### 2.1. Synthesis and Characterization of 4armPMB

The 4-functional-type initiator (pentaerythritol tetrakis(2-bromoisobutyrate):4f-BiB) was successfully synthesized by ester formation of pentaerythritol and 2-bromoisobutyryl bromide (Scheme S1).

To synthesize the 4-armed-type polymer (4-armPMB), MPC and BMA were copolymerized by ARGET-ATRP using 4f-BiB as an initiator. A linear-type polymer (LinearPMB) was synthesized by ARGET-ATRP using ethyl 2-bromoisobutyrate as the initiator. Table S1 summarizes the reaction conditions, and Table 1 summarizes the synthesis results for the polymers.

**Figure 2:**
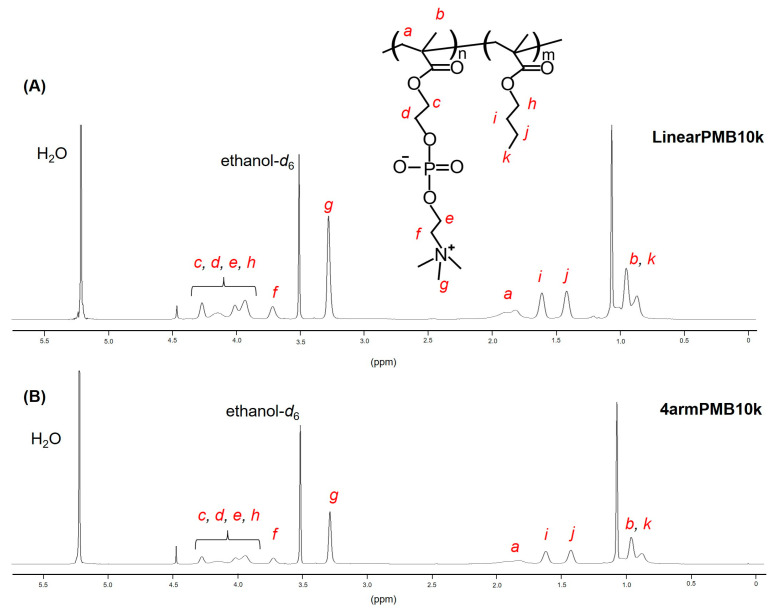
^1^H-NMR spectra of (**A**) LinearPMB10k and (**B**) 4armPMB10k (600 MHz, ethanol-*d*_6_). Each proton of the polymer was assigned to *a*–*k*.

The ^1^H-NMR spectrum (Figure 2) shows that methyl protons of the choline methyl group have a peak of 3.2−3.3 ppm (*g* peak in Figure 2), and methylene protons of butyl group have a peak of 1.3−1.7 ppm (*i* and *j* peaks in Figure 2). The copolymerization composition of the polymers corresponded to the monomer in feed. The SEC results indicated that the Mw of 4armPMB was greater than that of LinearPMB. This result suggests that the 4-armed-type polymer exhibited a larger excluded volume than the linear-type polymer because the core and polymer chain were restrained. Except for the MPC homopolymer, the 4armPMB and LinearPMB showed relatively narrow polydispersity indices (Mw/Mn) (1.3–1.6); therefore, polymerization depended on the living polymerization mechanism. The 4armPMPC exhibited Mw/Mn ≥ 2 (2.36). This wide range of Mw/Mn values may be caused by the high reactivity of MPC for polymerization. In addition, the 4armPMPC revealed a much greater Mw than that of 4armPMB. The polymer chain of Poly(MPC) in water is stretched and rigid according to an analysis of molecular simulation [30]. This is because the phosphorylcholine group is hydrophilic and extremely bulky compared with methacryloyl group. The poly(MPC) is assumed to be a rod-shaped polymer chain with high rigidity. The high rigidity of the poly(MPC) chain could have caused it to exhibit a high excluded volume to obtain high Mw. The hydrodynamic diameter and ζ-potential of polymers in aqueous solution indicated 4-armPMB and LinearPMB 15–30 nm and were almost neutral because the MPC unit canceled out its electric charge forming the internal salt between the phosphate and choline group.

**Figure 3:**
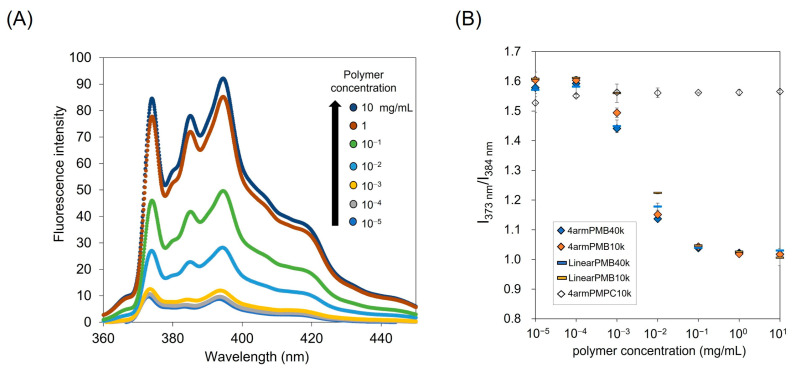
(**A**) Representative pyrene spectra in the presence of 4armPMB10k in PBS(−). The pyrene concentration was 1 mM, and emission spectra were in the range of 360 nm–450 nm (excitation wavelength: 337 nm) at 20 °C. (**B**) The ratio of pyrene fluorescence intensity (I_1_/I_3_) for the evaluation of the hydrophobic domain formation in a polymer aqueous solution.

The formation of the hydrophobic domain in phosphate-buffered saline (PBS(−)) was evaluated using a hydrophobic fluorescent probe, pyrene. Pyrene responds to the hydrophobicity of the environment and changes its fluorescence spectrum [31,32]. The fluorescence spectrum of pyrene in the polymer solution is shown in Figure 3. The fluorescence intensity increased in relation to the polymer concentration, and a high polymer concentration increases the dielectric constant of the solvent. The fluorescence spectrum of pyrene exhibits specific peaks in response to hydrophobicity. The fluorescence intensity ratio of I_1_ (373 nm) to I_3_ (384 nm) (I_1_/I_3_) was plotted against the polymer concentration, and the polymer without a BMA unit (4armPMPC10k) showed a constant value of I_1_/I_3_. The 4armPMPC exhibited high hydrophilicity and existed as a unimer because of the absence of a hydrophobic domain. In contrast, the I_1_/I_3_ of the 4armPMB and LinearPMB decreased as the polymer concentration increased. The polymer with BMA units formed a hydrophobic domain at greater than 10^−2^ mg/mL. These results indicated that 4armPMB and LinearPMB formed aggregations of polymers by hydrophobic interaction. No differences were observed between the 4-armed- and linear-type PMB in the formation of polymer aggregates. The content of the hydrophilic and hydrophobic units predominantly affected the formation of the aggregates. In addition, the molecular weights of the polymers showed no effect in the range of this study. The 4armPMB and LinearPMB revealed amphiphilicity in aqueous solutions such as PBS(−).

**Figure 4:**
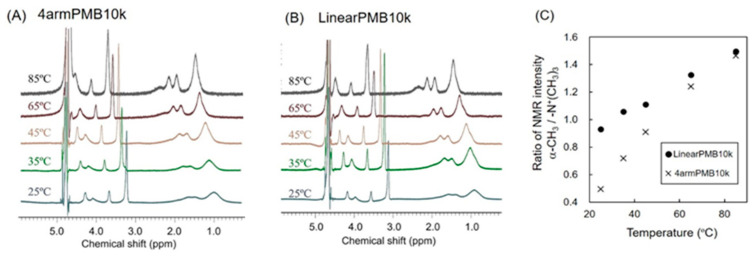
Temperature-dependent change in mobility of polymer chains.^1^H-NMR (600 MHz) spectra of (**A**) 4armPMB10k or (**B**) LinearPMB10k were obtained as a function of temperature in D_2_O. The concentration of polymer was 1 mg/mL. (**C**) The ratio of integration of NMR signal between choline methyl groups (-N^+^(CH_3_)_3_) in the side chain of MPC unit and α-methyl groups of the polymer backbone.

To estimate the mobility of the hydrophobic moiety of the polymer aggregates in the water, ^1^H-NMR spectra of polymers in D_2_O were obtained under various temperatures (Figure 4A,B). The changes in ^1^H-NMR spectrum in response to temperature increase are helpful for investigating the state of polymers aggregates as previously reported [32]. The peak at 1.0–1.5 ppm and 3.0–3.5 ppm were assigned to α-methyl protons of the polymer backbone and methyl protons of phosphorylcholine unit (-N^+^(CH_3_)_3_), respectively. Every peak derived from the polymer was shifted to the low magnetic field dependent on temperature increase. The intensity of 1.0–1.5 ppm signal increased at the higher temperature. These phenomena suggested that the mobility of the polymer chain was promoted due to a decrease in the viscosity of the solution likewise previously reported [32]. Ratios of NMR signal intensity between (-N^+^(CH_3_)_3_) and a-CH_3_ are shown in Figure 4C. LinearPMB indicated higher values than that of 4armPMB. The result suggested that the polymer aggregates of 4armPMB were more completely covered with MPC unit than the polymer aggregates of LinearPMB. Additionally, LinearPMB exhibited higher NMR signals (1.7–2.0 ppm) derived from BMA units than 4armPMB. These results suggested that the hydrophobic moiety of LinearPMB tended to locate the interface between the water and polymer aggregates. Furthermore, 4armPMB exhibited a higher ratio increase per temperature than LinearPMB. Therefore, star-shaped phospholipid amphiphilic polymers may easily change their conformation in response to temperature change. 

### 2.2. Cytotoxicity of 4armPMB and LinearPMB

**Figure 5:**
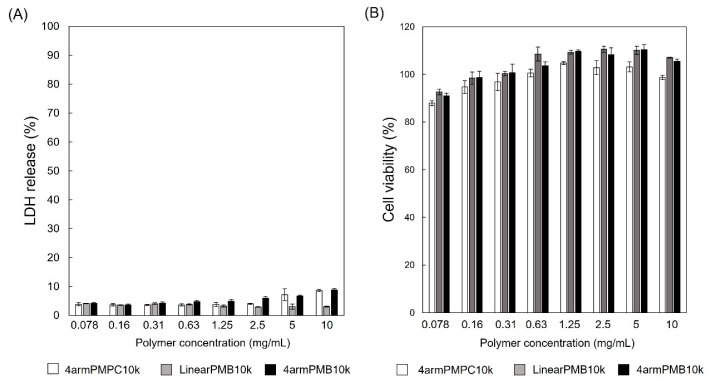
(**A**) LDH release of HeLa cells incubated with a 4armPMPC, 4armPMB10k, or LinearPMB10k aqueous solution for 24 h in DMEM with fetal bovine serum (5% FBS) at 37 °C, 5% CO_2_. The amount of LDH in the cell media was measured with an LDH assay kit. Then, 100% of LDH release was determined using 1% of Triton-X treated cells. (**B**) Cell viability of HeLa cells incubated with a polymer aqueous solution for 24 h in DMEM with 5% FBS. Cell viability was evaluated with a WST-8 assay.

The amphiphilic polymers, PMBs, have been reported to cause no cytotoxicity to cells. To estimate cytotoxicity and cell membrane damage from LinearPMB and 4armPMB synthesized by ARGET-ATRP, HeLa cells were incubated with polymers, and LDH and WST-8 assays were performed. The results of the LDH assay are shown in Figure 5A. The release of LDH from the cells was lower than 10% in cells treated with any of the polymers. The cell viability determined by the WST-8 assay (Figure 5B) was more than 80% in the range of 0.078–10 mg/mL. The 4armPMPC, 4armPMB10k, and LinearPMB10k showed no cytotoxicity or cell membrane damage, and they exhibited excellent cytocompatibility.

### 2.3. Cellular Uptake of Polymers

**Figure 6:**
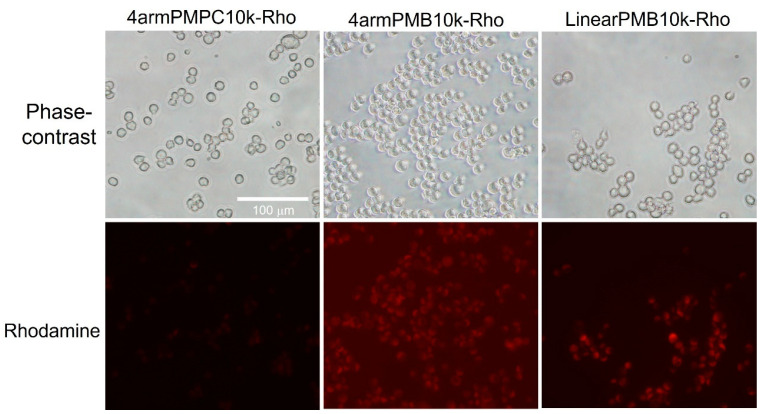
Phase-contrast and fluorescence images of DC2.4 cells incubated with rhodamine-labeled polymer (containing 0.1 mol% of rhodamine B unit) for 15 min in RPMI-1640 supplemented with FBS at 37 °C, 5% CO_2_. After incubation, cells were washed with HBSS, and the cells in HBSS were observed by fluorescence microscopy.

Figure 6 shows fluorescence microscopic images of DC2.4 cells incubated with rhodamine-labeled polymers. The 4armPMPC10k-Rho exhibited low cellular uptake because it is an MPC homopolymer, shown to have a lower cellular uptake in a previous study [22]. In contrast, in amphiphilic PMB polymers (4armPMB10k-Rho and LinearPMB-Rho), the fluorescence of rhodamine was observed in cells. This result showed that 4armPMB10k and LinearPMB10k showed cellular internalization over a short incubation time (15 min). These polymers could penetrate cell membranes based on previous reports [22]. From these data, it was suggested that the penetration and crossing of the cell membrane was more readily performed by 4armPMB than by LinearPMB10k-Rho. Figure S2 shows confocal microscopic images of DC2.4 cells treated with fluorescein-labeled polymers. These images show 4armPMB and LinearPMB located in cytosol due to green fluorescence from polymers situated in the different positions from lysosomes. This observation supported that PMB polymers were internalized by non-endocytic pathway. In addition, 4armPMB10k-Rho showed higher background fluorescence than that of LinearPMB10k-Rho. This phenomenon suggested that 4armPMB10k-Rho was exiting from inside the cell.

**Figure 7:**
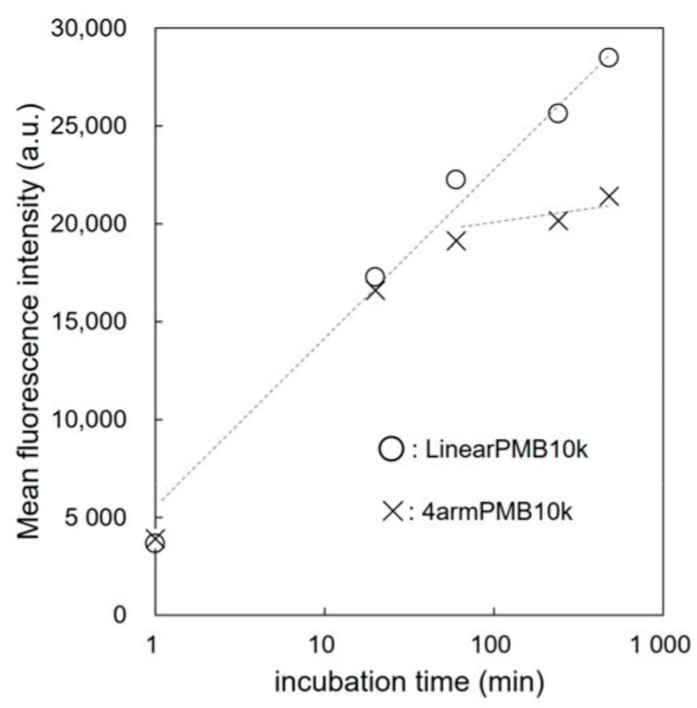
Incubation time-dependent cellular uptake of polymers. DC2.4 cells were incubated with rhodamine-labeled polymer (1 mg/mL) for the scheduled time in RPMI-1640 with FBS. Cells were then washed with HBSS, and the fluorescence intensity of cells was analyzed with a flow cytometer.

We quantitatively investigated the cellular uptake of the polymers using flow cytometry. The mean fluorescence intensity of approximately 10^4^ cells treated with rhodamine-labeled polymers is plotted against the incubation time in Figure 7. The 4armPMB10k-Rho and LinearPMB10k-Rho had the same fluorescence intensities at 1 and 60 min, which suggests that both polymers percolated through the cell membrane through their concentration gradient. After 60 min, the cellular uptake of LinearPMB10k-Rho increased, whereas that of 4armPMB10k-Rho plateaued, which indicates that the rate of cellular uptake of 4armPMB10k-Rho was balanced by the rate of diffusion to the outside of the cells. Rhodamine B-labeled PMB polymers have been reported to localize in the mitochondria because the rhodamine unit has an affinity for these organelles [33]. Thus, LinearPMB10k-Rho could have a stronger affinity to mitochondria than 4armPMB10k-Rho because LinearPMB10k-Rho is string shaped and can easily entangle in mitochondria. The star-shaped 4armPMB10k-Rho polymer has a branching point, which precludes it from readily entangling in mitochondria, and the core of the polymer has a large excluded volume effect. This consideration was not in conflict with the result of Figure 4C. LinearPMB10k-Rho aggregates may be easy to entangle to mitochondria because they expose their many hydrophobic units to the water phase.

**Figure 8:**
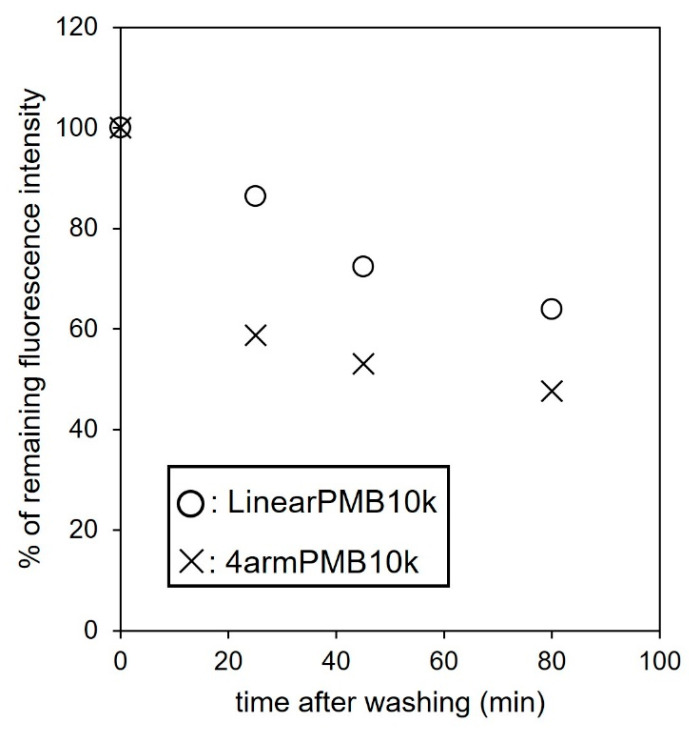
Back-diffusion of polymers internalized in DC2.4 cells. The remaining fluorescence in the cells was detected by flow cytometry. The fluorescence intensity of cells treated with rhodamine-labeled polymer for 60 min was regarded as 100% of remaining fluorescence intensity.

Finally, back-diffusion of the polymer from the cells was evaluated, as shown in Figure 8. LinearPMB10k-Rho and 4armPMB10k-Rho diffused from the cell depending on the incubation time, while 4armPMB10k-Rho escaped more quickly than LinearPMB10k-Rho from the cells. This result suggests that the aggregates of the star-shaped polymer could be easily passing through the cellular membrane because MPC units covered the polymer aggregates. Thus, the architecture of the polymer affects cellular internalization and exiting.

## 3. Materials and Methods

### 3.1. Materials

MPC was purchased from Sigma-Aldrich Japan Co., LLC. (Tokyo, Japan). BMA, pentaerythritol, and triethylamine were purchased from FUJIFILM Wako Pure Chemical Corporation (Osaka, Japan), while 2-Bromoisobutyryl bromide was obtained from Tokyo Chemical Industry Co., Ltd. (Tokyo, Japan). Ethylene bis(2-bromobutyrate), 2,2′-bipyridyl, copper(II) bromide, and l-ascorbic acid were purchased from Sigma-Aldrich Japan Co., LLC. (Tokyo, Japan). HeLa cells were obtained from the RIKEN Cell Bank (Saitama, Japan). RPMI-1640, Dulbecco’s modified Eagle medium (DMEM), fetal bovine serum (FBS), and phosphate-buffered saline (PBS) were purchased from Thermo Fisher Scientific, Inc. (Waltham, MA, USA). Cell counting kit-8 (CCK8) was purchased from Dojindo Laboratories Co., Ltd. (Kumamoto, Japan), LDH-Cytotoxic Test Wako from FUJIFILM Wako Pure Chemical Corporation (Osaka, Japan), Spectra/Por dialysis membrane from Spectrum Chemical Mfg. Corp. (New Brunswick, NJ, USA), and Methacryloxyethyl thiocarbamoyl rhodamine B from Polysciences Inc. (Warrington, PA, USA). The other solvents and reagents were of extra-pure grade and used without further purification.

### 3.2. Synthesis of Pentaerythritol Tetrakis(2-bromoisobutyrate) (4f-BiB)

The tetrafunctional-type initiator, pentaerythritol tetrakis(2-bromoisobutyrate), was synthesized as previously reported [34]. Briefly, pentaerythritol (3.115 g, 22.9 mmol) was added to a flask, which was purged with argon gas. Dichloromethane anhydrous (DCM) (70 mL) was added as a solvent and triethylamine (13.5 mL, 97.0 mmol, 1.1 eq of OH) was included and stirred in an ice bath; 2-Bromoisobutyrate bromide (12.5 mL, 97 mmol, 1.1 eq of OH) was dissolved in 10 mL of DCM, and the solution was added dropwise into the flask. The reaction mixture was stirred for 3 h in an ice bath and then stirred for 12 h at around 20 °C. The resulting solution was washed with distilled water three times and saturated with NaHCO_3_ aq. The organic phase was dried with Na_2_SO_4_ and then filtered, and the filtrate was concentrated using a rotary evaporator. The solution was purified by recrystallization twice with methanol as the poor solvent to afford white needle-shaped crystals, with a yield of 44.9%. The ^1^H-NMR spectrum is shown in Figure S1.

### 3.3. Synthesis of Polymers

The 4-armed-type and linear-type polymers were synthesized by ARGET-ATRP. The 4-armed-type initiator, 4f-BiB (50 mg, 0.0683 mmol) was dissolved in DMF (1 mL), while MPC (1.11 g, 3.75 mmol) and BMA (1.24 g, 8.75 mmol) were dissolved in ethanol (19 mL). To synthesize the linear-type polymers, 2,2′-bromoisobutyrate was dissolved in ethanol. The initiator and monomer solutions were mixed in a round-bottomed flask, and 2,2′-Bipyridyl (bpy) (53 mg, 0.34 mmol) and copper(II) bromide (1.5 mg, 6.8 mol) were added to produce a light green solution. An l-ascorbic acid (96 mg, 0.54 mmol) was then added to the flask. The solution was purged with argon gas for 10 min and the flask was sealed with a glass stopcock. The color of the solution turned brown. The flask was soaked in an oil bath at 60 °C, and the reaction solution was stirred for 24 h. The resulting solution was then reprecipitated using a mixture of chloroform and diethyl ether (1/9, v/v) to obtain a light brown solid, which was dialyzed against water for three days using a dialysis membrane (MWCO: 3.5 kDa). Subsequently, the product was lyophilized to obtain a white solid. The copolymer compositions were determined by ^1^H-nuclear magnetic resonance (^1^H-NMR) using ethanol-*d_6_* as the solvent. The weight-averaged and number-averaged molecular weights (Mw and Mn, respectively) were estimated by size exclusion chromatography (SEC). The OHpak 803-HQ (Showa Denko, Kawasaki, Japan) was used as the column. A mixture of methanol and water (7/3, v/v) with 10 mM LiBr was used as the eluent at a flow rate of 0.5 mL/min. The polymer signal was detected using the refractive index (RI) detector RI-4030 (JASCO Corp., Tokyo, Japan). A calibration curve was prepared using polyethylene oxide as the standard. The ^1^H-NMR spectrum is shown in Figure 2. The characterization of the polymers is summarized in Table 1.

### 3.4. Evaluation of the Hydrophobic Domain in the Polymer Solution

To determine the critical aggregation concentration of the polymer in an aqueous solution, pyrene was used as a fluorescent probe [31]. The pyrene in acetone solution was placed in a glass vial, and the acetone was evaporated under a vacuum. The polymer was dissolved in phosphate-buffered saline, PBS(−), at 10^−5^–10^1^ mg/mL and the polymer solution was added to the glass vial and incubated overnight. The final concentration of pyrene was 1 μM. The fluorescence spectra were measured using a spectrofluorophotometer at an excitation wavelength of 337 nm (FP-6500, JASCO Corp., Tokyo, Japan).

### 3.5. Hydrodynamic Diameter of Polymers

The obtained polymer was dissolved in 1 mM Na_2_HPO_4_ (pH 7.4) at a concentration of 1 mg/mL. The hydrodynamic diameter was measured using with dynamic light scattering (DLS). ζ-potential was measured using laser-Doppler electrophoresis (ZetasizerPro, Malvern Panalytical Ltd., Malvern, UK).

### 3.6. Evaluation of Polymer Chain Mobility

The polymer was dissolved in D_2_O at a concentration of 1 mg/mL. ^1^H-NMR spectra at various temperature conditions were obtained by JNM ECA-600 (JEOL Ltd., Tokyo, Japan).

### 3.7. Observation of Cellular Penetration

Rhodamine-labeled polymers were synthesized using methacryloxyethyl thiocarbamoyl rhodamine B (0.1 mol% of monomers), as described above. HeLa cells (1 × 10^5^ cells) were seeded in a 12-well dish and pre-incubated for 24 h in DMEM in the presence of serum (10% FBS). Cells were washed twice with Hanks’ balanced salt solution (HBSS). Subsequently, 1 mL of fresh DMEM with serum was added to the dish along with the rhodamine-labeled polymer solution in PBS(−), and cells were incubated for 15 min at 37 °C and 5% CO_2_. The final polymer concentration was 1 mg/mL. After incubation, the cells were washed with HBSS three times, and 1 mL of HBSS was added to the dish. Cells were then observed under a fluorescence microscope (Research inverted system microscope IX71, Olympus, Tokyo, Japan).

### 3.8. Cytotoxicity Measurement

HeLa cells (1 × 10^4^ cells) were precultured in a 96-well dish for 24 h. The cells were then washed twice with HBSS. DMEM (100 μL) with 10% serum was added to the wells along with the polymer solution in PBS (100 μL). The cells were incubated for 24 h at 37 °C and 5% CO_2_. After incubation, cell viability and cell membrane damage were evaluated using the Cell Counting kit-8 and LDH-assay kits, respectively.

### 3.9. Flow Cytometric Analysis of Cellular Uptake

A mouse dendritic cell line, DC2.4 cells (1 × 10^5^ cells) was precultured for 24 h in RPMI-1640 supplemented with 10% FBS. The cells were washed twice with HBSS, and RPMI-1640 (1 mL) with serum was added to the wells. Rhodamine-labeled polymer solution in PBS was then added, and the cells were incubated for a prescheduled time in the incubator at 37 °C and 5% CO_2_. After incubation, the cells were washed with HBSS twice and then detached using trypsin-EDTA solution. The fluorescence intensity of the cells was analyzed by flow cytometry (CytoFlex, Beckman Coulter, Inc., Brea, CA, USA).

### 3.10. Statistical Analysis

Data are expressed as means ± standard deviation (SD). Significance levels were set at ** (*p* < 0.01) and * (*p* < 0.05) using the Student’s *t*-test.

## 4. Conclusions

**Figure 9:**
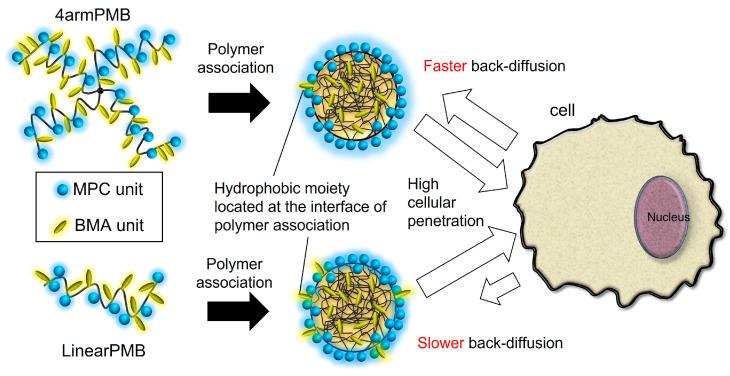
Schematic illustration of 4-armed and linear PMB polymers interacting with cells. The association of 4armPMB polymer was almost covered with MPC unit. The association of LinearPMB was covered with MPC and BMA units. The back-diffusion behavior of 4armPMB polymer aggregates was faster than LinearPMB.

The 4-armed branched star-shaped and linear-type polymers were synthesized via ARGET-ATRP. The monomers MPC and BMA were copolymerized to obtain 4armPMB and LinearPMB. These polymers exhibited similar amphiphilicities as determined using the fluorescence probe pyrene. However, ^1^H-NMR spectra measured in D_2_O revealed that 4armPMB aggregates tended to change their conformation in response to temperature increase compared with LinearPMB. Both 4armPMB and LinearPMB did not damage the cell membrane, exhibited excellent cytocompatibility, and were taken up by cells within a short exposure time. Furthermore, 4armPMB and LinearPMB entered and departed the cells, which corroborates previous reports on PMB polymers. The 4armPMB showed a higher rate of escape from cells than that of the LinearPMB. The star-shaped polymer exhibited different behaviors of cellular uptake and escape than those of the linear-shaped polymer. Figure 9 summarizes the difference between 4armPMB and LinearPMB. It was slightly easy for 4armPMB to escape from cells because it may be difficult for the aggregates of 4armPMB covered with MPC units to interact with organelle in cells. The star-shaped amphiphilic polymers are potentially superior drug solubilizers with the function of sustained drug release. They can exhibit the characteristics of a “cell shuttle,” a delivery carrier that departs and returns. In future studies, star-shaped amphiphilic polymers can be applicable for structural materials in the biomedical field, such as hydrogels and surface-coating agents.

## Figures and Tables

**Table 1:** Characterization of polymers.

Abb.	Mw	Mw/Mn ^(a)^	Composition (mol%) ^(b)^		Hydrodynamic Diameter (nm) ^(c)^	ζ-Potential (mV)
			MPC	BMA		
4armPMB10k	14,000	1.39	30	70	25	−2.1 ± 0.5
4armPMB40k	37,000	2.14	30	70	19	−8.7 ± 3.1
LinearPMB10k	5400	1.22	30	70	15	−0.5 ± 0.2
LinearPMB40k	9600	1.55	30	70	14	−4.7 ± 0.2
4armPMPC10k	33,000	2.86	100	0	18 ^(d)^	−3.5 ^(d)^

^(a)^ determined by SEC (eluent: methanol/water, 7/3). ^(b)^ determined by ^1^H-NMR (solvent: ethanol-*d*_6_). ^(c)^ measured with DLS in 1 mM Na_2_HPO_4_aq at 25 °C. ^(d)^ measured at 10 °C.

## Supplementary Materials

The following supporting information can be downloaded at: https://www.mdpi.com/article/10.3390/molecules28114479/s1, Scheme S1: Synthesis of pentaerythritol tetrakis(2-bromoisobutyrate) (4f-BiB); Figure S1: ^1^H-NMR spectrum of pentaerithrytol tetrakis(2-bromoisobutyrate (4f-BiB); Figure S2: Confocal laser-scanning microscopic images; Table S1: The reaction conditions of polymerization.
