# Supplementary material for: Cellular Internalization and Exiting Behavior of Zwitterionic 4-Armed Star-Shaped Polymers
Source: Molecules. 2023 Jun 1;28(11):4479. doi: 10.3390/molecules28114479 (PMC10254323; doi:10.3390/molecules28114479)
Supplement: Supplementary file 1 [file molecules-28-04479-s001.zip › molecules-2395017-supplementary.pdf]

[Supporting Information]

Cellular Internalization and Exiting Behavior of Zwitterionic 4-Armed Star-Shaped Polymers

Yuta Yoshizaki and Tomohiro Konno *

Graduate School of Pharmaceutical Sciences, Tohoku University, Sendai 980-8578, Japan; yuta.yoshizaki.c5@tohoku.ac.jp
* Correspondence: t-konno@tohoku.ac.jp; Tel.: +81-22-795-6841

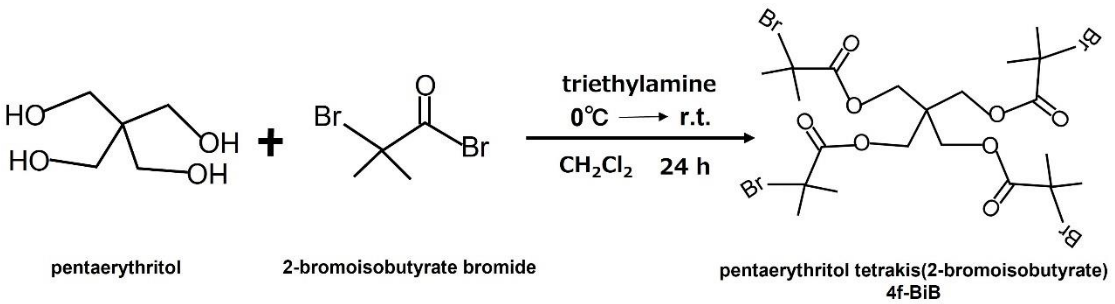

Scheme S1. Synthesis of pentaerythritol tetrakis(2-bromoisobutyrate) (4f-BiB).

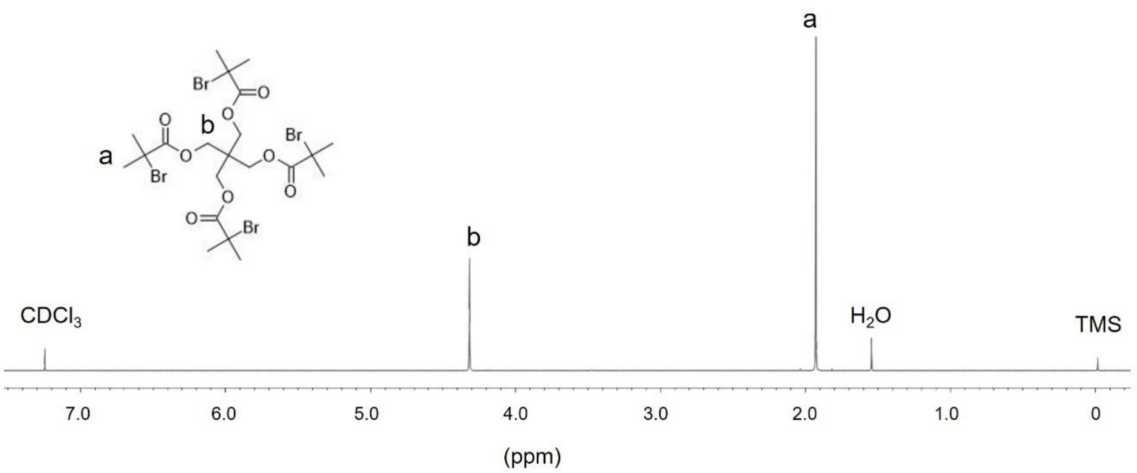

Figure S1. ^{1}H-NMR spectrum of pentaerithrytol tetrakis(2-bromoisobutyrate) 4f-BiB (600 MHz, $CDCl_3$).

Table S1. The reaction conditions of polymerization

Abb.	The Feed Ratio of [Monomer]/[Initiator]	Solvent
4armPMB10k	44	Ethanol/DMF (19/1, v/v)
4armPMB40k	177	Ethanol/DMF (19/1, v/v)
LinearPMB10k	44	Ethanol
LinearPMB40k	177	Ethanol
4armPMPC10k	36	Ethanol/DMF (19/1, v/v)

The feed ratio of [$CuBr_2$]/[Initiator] = 1/50. The feed ratio of [2,2'-bipyridyl]/[Initiator] = 1/1. The feed ratio of [Ascorbic acid]/[Initiator] = 10/1.

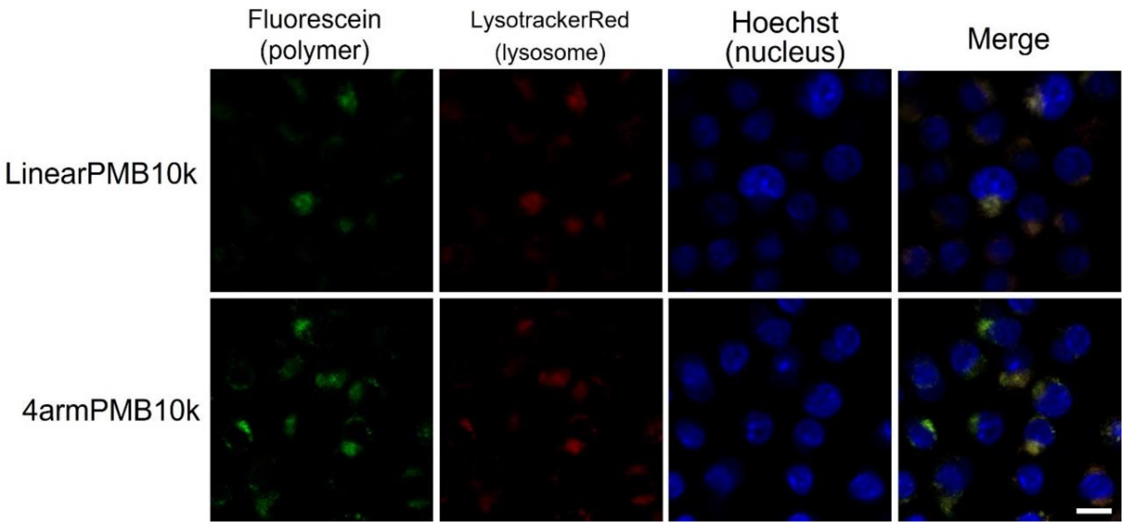

Figure S2. Confocal laser-scanning microscopic (CLSM) images of DC2.4 cells. Cells were incubated with 1 mg/mL of polymers in the presence of 10% FBS-supplemented RPMI-1640 for 6 h. Polymers were labeled with 0.1mol% of fluorescein methacrylate and cells were stained with LysotrackerRed (lysosome) and Hoechst33342 (nucleus). Scale bar represents 20 μm. Images were acquired by using Nikon C2 confocal microscope.
